# Implementation of an electronic fingerprint-linked data collection system: a feasibility and acceptability study among Zambian female sex workers

**DOI:** 10.1186/s12992-015-0114-z

**Published:** 2015-06-27

**Authors:** Kristin M. Wall, William Kilembe, Mubiana Inambao, Yi No Chen, Mwaka Mchoongo, Linda Kimaru, Yuna Tiffany Hammond, Tyronza Sharkey, Kalonde Malama, T. Roice Fulton, Alex Tran, Hanzunga Halumamba, Sarah Anderson, Nishant Kishore, Shawn Sarwar, Trisha Finnegan, David Mark, Susan A. Allen

**Affiliations:** Department of Epidemiology, Rollins School of Public Health, Laney Graduate School, Emory University, Atlanta, GA USA; Rwanda Zambia HIV Research Group, Department of Pathology and Laboratory Medicine, School of Medicine and Hubert Department of Global Health, Rollins School of Public Health, Emory University, 1518 Clifton Road NE, CNR 4011, Atlanta, GA 30322 USA; Rwanda Zambia HIV Research Group, Department of Pathology and Laboratory Medicine, School of Medicine and Hubert Department of Global Health, Rollins School of Public Health, Emory University, Lusaka, Zambia; Rwanda Zambia HIV Research Group, Department of Pathology and Laboratory Medicine, School of Medicine and Hubert Department of Global Health, Rollins School of Public Health, Emory University, Ndola, Zambia; Gavi, the Vaccine Alliance, Geneva, Switzerland; Department of Public Health Sciences, Karolinska Institutet, Stockholm, Sweden; Biometrac, Washington, DC USA; Biometrac, Louisville, KY USA; International AIDS Vaccine Initiative, New York, NY USA

**Keywords:** Biometric identification, Fingerprinting, Female sex workers, HIV/AIDS, Patient care, Key populations, Stigmatized populations, Zambia

## Abstract

**Background:**

Patient identification within and between health services is an operational challenge in many resource-limited settings. When following HIV risk groups for service provision and in the context of vaccine trials, patient misidentification can harm patient care and bias trial outcomes. Electronic fingerprinting has been proposed to identify patients over time and link patient data between health services. The objective of this study was to determine 1) the feasibility of implementing an electronic-fingerprint linked data capture system in Zambia and 2) the acceptability of this system among a key HIV risk group: female sex workers (FSWs).

**Methods:**

Working with Biometrac, a US-based company providing biometric-linked healthcare platforms, an electronic fingerprint-linked data capture system was developed for use by field recruiters among Zambian FSWs. We evaluated the technical feasibility of the system for use in the field in Zambia and conducted a pilot study to determine the acceptability of the system, as well as barriers to uptake, among FSWs.

**Results:**

We found that implementation of an electronic fingerprint-linked patient tracking and data collection system was feasible in this relatively resource-limited setting (false fingerprint matching rate of 1/1000 and false rejection rate of <1/10,000) and was acceptable among FSWs in a clinic setting (2 % refusals). However, our data indicate that less than half of FSWs are comfortable providing an electronic fingerprint when recruited while they are working. The most common reasons cited for not providing a fingerprint (lack of privacy/confidentiality issues while at work, typically at bars or lodges) could be addressed by recruiting women during less busy hours, in their own homes, in the presence of “Queen Mothers” (FSW organizers), or in the presence of a FSW that has already been fingerprinted.

**Conclusions:**

Our findings have major implications for key population research and improved health services provision. However, more work needs to be done to increase the acceptability of the electronic fingerprint-linked data capture system during field recruitment. This study indicated several potential avenues that will be explored to increase acceptability.

## Introduction

Patient identification in healthcare settings ensures accuracy of collected data and enhances patient care [1]. Patient misidentification contributes substantially to medication, testing and referral errors, resulting in poorer health outcomes [2–5] and costs healthcare systems billions of dollars each year [6]. The Institute of Medicine estimates people are 2.6 times more likely to die of patient identification errors than motor vehicle accidents [7]. A 2011 World Health Organization report named patient identification one of nine priority Patient Safety Solutions globally [1] with specific relevance in sub-Saharan Africa [8].

Patient identification within and between health services is an operational challenge in much of sub-Saharan Africa [9, 10]. A survey in 30 health facilities in Rwanda, Burundi, Mali, Ivory Coast, and the Democratic Republic of Congo found 93 % of health management teams reported major challenges with patient identification [9]. A study of six health facilities in Rwanda and Burundi found that patient misidentification occurred in 65 % of 1396 patient visits [9]. A South African study found that errors were relatively infrequent when reporting patient names (6 % of cases) and very high when recording patient folder numbers (in 65 % of cases) in a clinical setting [10]. A study in Malawi found 34 % of hospital staff reported a misidentification event per year, and only 6 % of staff used identifiers other than name [11].

Current identification methods in sub-Saharan Africa do not confirm identity. Most health clinics in sub-Saharan Africa use patient name, date of birth, government ID, or phone number to identify patients, and record numbers are not centralized, making patients virtually impossible to follow across services [9]. Moreover, these identifiers are often inexact in the African context: a study of 27 Rwandan and Burundian health facilities found many patients do not know their exact date of birth, and patient names and spellings varied. As a result, multiple medical record numbers existed for given patients within the same facility [9].

Additionally, use of names raises confidentiality concerns, which is a common barrier to HIV testing and prevention access [12–18], particularly among vulnerable populations [19–22]. For example, stigma and discrimination associated with sex work or being sexually active outside of wedlock have been reported as barriers to providing identifiers to health workers (name, date of birth, phone number, government ID numbers) in order to access HIV prevention and treatment services [23, 24]. HIV prevention for vulnerable populations that promotes equal access to health services and respect of confidentiality are key public health priorities [25].

Currently, the Rwanda Zambia HIV Research Group (RZHRG) is enrolling HIV negative female sex workers (FSWs) in a longitudinal study in Zambia in preparation for a mock vaccine trial. These populations are at high risk of HIV acquisition in sub-Saharan Africa [26] where annual incidence ranges from 4-11 % for FSWs [27–30]. HIV prevalence among Zambian FSWs is estimated at 65-69 % [31, 32]. The goals of this study are to 1) assess HIV risk factors to improve HIV prevention and 2) estimate HIV incidence to determine suitability of FSWs for future HIV vaccine trials. The latter issue is key for prevention or intervention trial preparedness: a recent systematic review observed HIV incidence was lower than expected in 73 % (8/11) of such trials [33], and two microbicide trials were halted due to low HIV incidence [34, 35]. It is thought that trial incentives may lead to patient fraud [36] leading to lower HIV risk in the cohort. For example, patients in Cambodia fraudulently used tuberculosis treatment cards to obtain incentives [37], and unpublished data from IAVI revealed individuals from cohorts of men who have sex with men were co-enrolled in the same study at two centers (Nairobi and Kilifi), and cross enrollment occurred in clinical trials in Durban (personal communication, Dr. Pat Fast and David Mark, IAVI). RZHRG currently relies on matching phone numbers of FSWs recruited in the field with those who enroll, which does not confirm identity [19]. After enrollment, identity is verified by matching ink fingerprints across follow-up visits which, though acceptable to >99 % of clients, is time-consuming and highly prone to error [38].

Pato et al., in a review for the National Academy of Sciences, noted that biometric systems are used increasingly to improve the efficiency of transactions and reduce fraud. However, these systems were also found to be “inherently probabilistic”, with any weaknesses in the systems or operational design negatively impacting the validity of patient matching. Successful deployments were found to deal with issues such as security, local applicability and scalability with good project management, adequate oversight, locally pertinent biometric interventions and adaptive technologies that kept pace with advancements in the field [39]. In this context, electronic fingerprinting has been proposed to identify patients over time and link patient data between health services [1, 40]. This simple, inexpensive technology is user-friendly, cellular-based, and portable, overcoming key challenges in resource-limited settings. Furthermore, there is stakeholder and donor support for this technology. The World Health Organization recommends implementation of standardized identification approaches, including biometric technologies in health settings [1, 41–43]. Though still in nascent stages, these technologies are being tested for patients in the United States under a National Institutes of Health (NIH)-funded grant to reduce medical errors due to misidentification [44]. In South Africa, researchers conducted a pilot acceptability study and found high (>90 %) adult electronic fingerprint acceptability (i.e., non-refusal) rates among non-stigmatized populations [45, 46]. Another group in South Africa successfully used fingerprints as their patient identifier among 1130 clients enrolled in a mobile HIV testing and active tuberculosis case finding program [47]. A patient identification system using electronic fingerprinting developed by Vaxtrac/Biometrac (Gates Grand Challenge winners) successfully tracked vaccination schedules among children in India and Benin [48, 49]. This system often required validation of identity with the fingerprint of the mother due to numerous challenges in processing infant fingerprints, however, refinements in the Vaxtrac/Biometrac fingerprinting algorithm tested by Jain et al. showed that the best results could be obtained from a fusion of match scores from two fingers from the child [50]. Importantly, as the software and hardware for fingerprint matching has historically, been designed for adults, match rates for the post-adolescent were notably higher (up to 99 %). Finally, RZHRG recently completed a pilot study demonstrating the technical feasibility of the Biometrac fingerprinting system among couples’ HIV testing, antiretroviral treatment, family planning, and male circumcision clients in Zambia.

While electronic fingerprinting systems continue to grow in popularity, the feasibility and acceptability of such a device among vulnerable or stigmatized populations is largely unknown. A study in Rwandan government health facilities found patients expressed concern about how their fingerprints could be used by the government [9]. Conversely, the Desmond Tutu Foundation has recently implemented electronic fingerprinting of patients within mobile HIV testing units to *increase* patient confidentiality and anonymity where it was noted that stigmatized patients would rather provide a fingerprint than have their name recorded or used publicly [51]. Though this idea may be contrary to common concepts of fingerprinting and confidentiality, it is virtually impossible to trace or locate an individual using a fingerprint in countries with no nationally identifiable fingerprint database. Initial pilot testing by RZHRG found that device acceptance among FSWs when approached in their work location was low. In this paper, we detail the feasibility, including technical challenges, with implementing an electronic-fingerprint linked data capture system in clinics in Zambia, and the acceptability and barriers to uptake of this system among FSWs.

## Methods

### The device/system

**Fig. 1:**
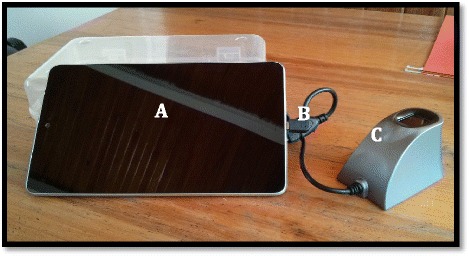
Electronic fingerprint-linked data collection system. **a**. Android touchscreen tablet for data input. **b**. USB adaptor cable. **c**. Single-finger imaging sensor for fingerprint collection

**Fig. 2:**
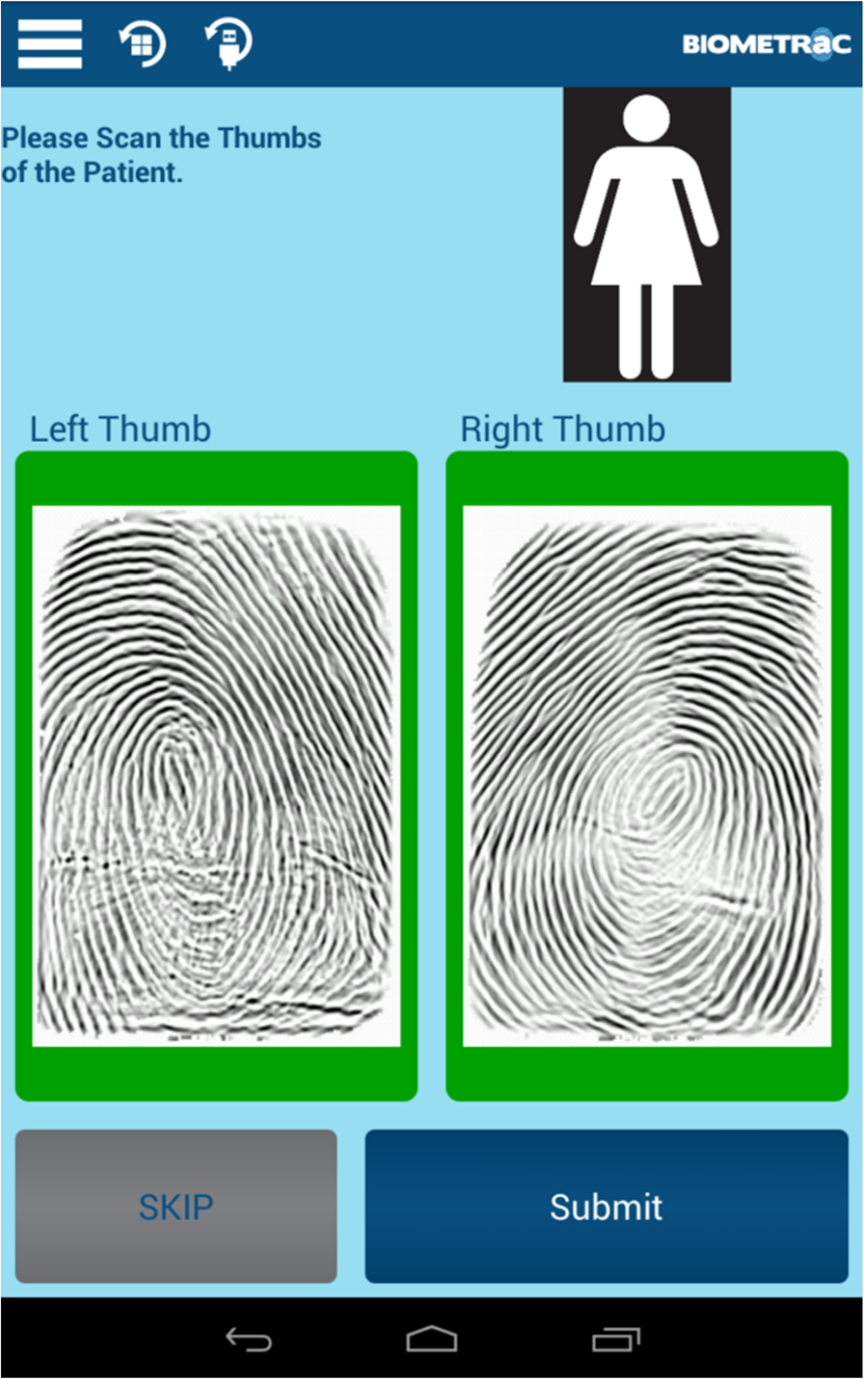
Tablet screenshot of scanned fingerprints for female clients

IAVI and RZHRG entered into a contract with Biometrac [48, 49] and purchased 38 electronic fingerprint scanning and data collection systems which consist of a Google Nexus 7 tablet with a portable single-finger multi-spectral imaging sensor connected via USB (Fig. 1). Each device has security software for real-time tracking and clearing if stolen. The contract includes technical design, software customization, on-site training and testing, master database security and maintenance, and annual site licenses. The device scans both thumbs and index fingers (Fig. 2) and transmits encrypted templates and inputted data to a central server via Global System for Mobile (GSM). Mobile coverage in Zambian urban and rural areas is relatively high, with the GSM network operated by Zambian carrier MTN covering ¾ of the population and increasing [52]. Records are collected in a database viewable on a password-secured website. Service-specific data entry workflows for FSW recruitment and clients of HIV testing, family planning, male circumcision, and antiretroviral treatment services have been developed. These workflows capture staff initials, clinic name, service type, and fingerprints.

### The training protocol

A half day training for device users includes a didactic component, post-test assessment, practical component, and practical skills assessment. The didactic component describes the overall goal of and rationale for implementing the system, why the device is more effective than current methods, device uses and potential adaptability based on user needs, security issues, good fingerprinting technique, device system startup, step-by-step use of all workflows, troubleshooting common problems, and how to report system errors and client refusals. “Workflows” are the series of data input screens that the system user is prompted with. This training includes a description of the protocol for approaching clients before asking them to provide fingerprints. Staff inform clients about why we are using the fingerprinting system, explain how the system is more efficient relative to other methods, and clarify that client participation is voluntary and that their information is confidential. A post-test quiz assesses user knowledge of training themes. The practical training is structured so that each user practices fingerprinting with two other individuals four times, and all users practice system shut down, restart, and charging. After initial practice, the trainer cross-references data from the narratives with data accessed from a central online database storing all captured fingerprint records. The trainer retrains on areas where trainees did not accurately enter data. Trainees repeat entering narratives with a new partner. Trainees must score at least 80 % on the post-test quiz and achieve 100 % accurate data entry on the second round to be certified to use the device. We have also developed a quantitative survey that assesses user satisfaction with system and training issues.

### Pilot study feasibility, acceptability, and barriers to uptake

From May-August 2013, an initial assessment of device feasibility was conducted in four clinics in Ndola, Zambia. This included assessments of clinic infrastructure, cellular connection, security (specifically, having a locked location to charge the device at night), and staff technological competence. To measure device false positive and false negative matching rates, we fingerprinted a sample of RZHRG counselors and staff three times each in government clinics or under conditions similar to government clinics. We then used a receiver operating characteristics (ROC) plot of the false positive and false negative matching rates under different fingerprinting scenarios (e.g., thumbs only, index fingers only, both thumbs and index fingers) and thresholds (“threshold” is the cutoff point at which the algorithm will either accept or reject a fingerprint as a match) to determine the *equal error rate*, which is a value at which the false positive and false negative matching rates are equal. Thus, at the equal error rate, the proportion of false positives is equal to the proportion of false negatives; lower equal error rates indicate increased accuracy of the biometric system.

To determine the acceptability of the devices, FSWs were asked to voluntarily provide electronic fingerprints during in-field recruitment, screening, enrollment, and follow-up visits. Acceptability was defined as the number of women agreeing to provide an electronic fingerprint divided by the total number of women approached for fingerprinting. Fingerprinting at the FSW place of work was identified as a possible solution to eliminate “false FSW” – women who came to the research site claiming to be sex workers in order to get health benefits.

To determine the facilitators and barriers to voluntary electronic fingerprinting in the field, we used formative qualitative methods to inform the development of a quantitative survey. First, we conducted two focus groups with 10 FSWs. Two RZHRG FSW recruiters facilitated and took notes. Focus group discussion topics were pilot tested for cultural and educational appropriateness among five FSWs and five recruiters to improve clarity and content. During the focus groups, the device was explained to participants in terms of its uses, importance, and advantages relative to other patient identification methods, including the confidentiality of the system. Participants were shown the electronic fingerprinting system, given time to handle the device, and practice fingerprinting. Participants were then asked to discuss their initial impressions of the device and system. Focus group discussion topics related to facilitators and barriers to voluntary electronic fingerprinting in the field were guided by themes from preliminary findings. For example: *Do you think the system increases or decreases confidentiality? What are barriers/facilitators to being electronically fingerprinted while you are working/at antenatal care (e.g., confidentiality, location/privacy, the way the device is presented to you, the way recruiters approach you)? Would you be more likely to provide a fingerprint to an FSW recruiter (for FSW focus groups)? Would you be more likely to provide a fingerprint to a recruiter with a badge, who presented proof of clinic services?* The analysis of the focus group transcripts focused on identification of key response items using thematic analysis. In the thematic analysis framework, theme coding is constructed guided by experimental interests and relevant issues that arise in the transcripts. Our focus groups consisted of convenience samples, appropriate for qualitative analyses where it is recommended that at least 6–8 sampling units are often sufficient and 12–20 data sources preferred [53, 54].

The aim of this analysis was to identify themes that would be needed to include in the quantitative survey. The quantitative surveys were then designed to capture the following information regarding other places women would be willing to provide fingerprints: (1) the attitude toward providing electronic fingerprints in the field before and after receiving explanation to the system, (2) the preferred location for providing electronic fingerprints, (3) the utility of our proposed intervention for increasing acceptability, which includes recruiting women in small groups, providing incentives, and formalizing the recruitment process through the use of RZHRG badges and the involvement of Queen Mothers, who are women that represent and/or house one or more FSWs [55].

Surveys were delivered orally by nurse counselors at the Zambia Emory HIV Research Project (ZEHRP) study sites in Lusaka and Ndola. The surveys targeted the previously-screened HIV negative FSWs who were at least 18 years of age and were to be enrolled in ZEHRP’s HIV risk factor study. As the woman came to the ZEHRP study site for enrollment appointment, nurses consented them individually and then administered the oral survey in the women’s preferred languages including Nyanja and Bemba. All FSW enrollees were informed of the purpose of this survey via an oral informed consent and given freedom to terminate their participation at any time during survey administration. The nurses were encouraged to probe for clarification if the initial responses from the FSW participants were ambiguous. The nurses were also required to record the women’s response to each survey question, which were then de-identified and entered into an Excel datasheet. To ensure the quality of survey data, nurses were required to provide oral or written clarification if responses were inconsistent or missing. In addition, each de-identified survey was photo-scanned and stored electronically for future reference if needed. Upon completion of data collection, the Excel datasheet were imported to SAS 9.4, in which the survey data were analyzed as counts and percentages. This study protocol was reviewed and approved by the Emory IRB and the Zambia Research Ethics Committee.

## Results

### Feasibility

**Fig. 3:**
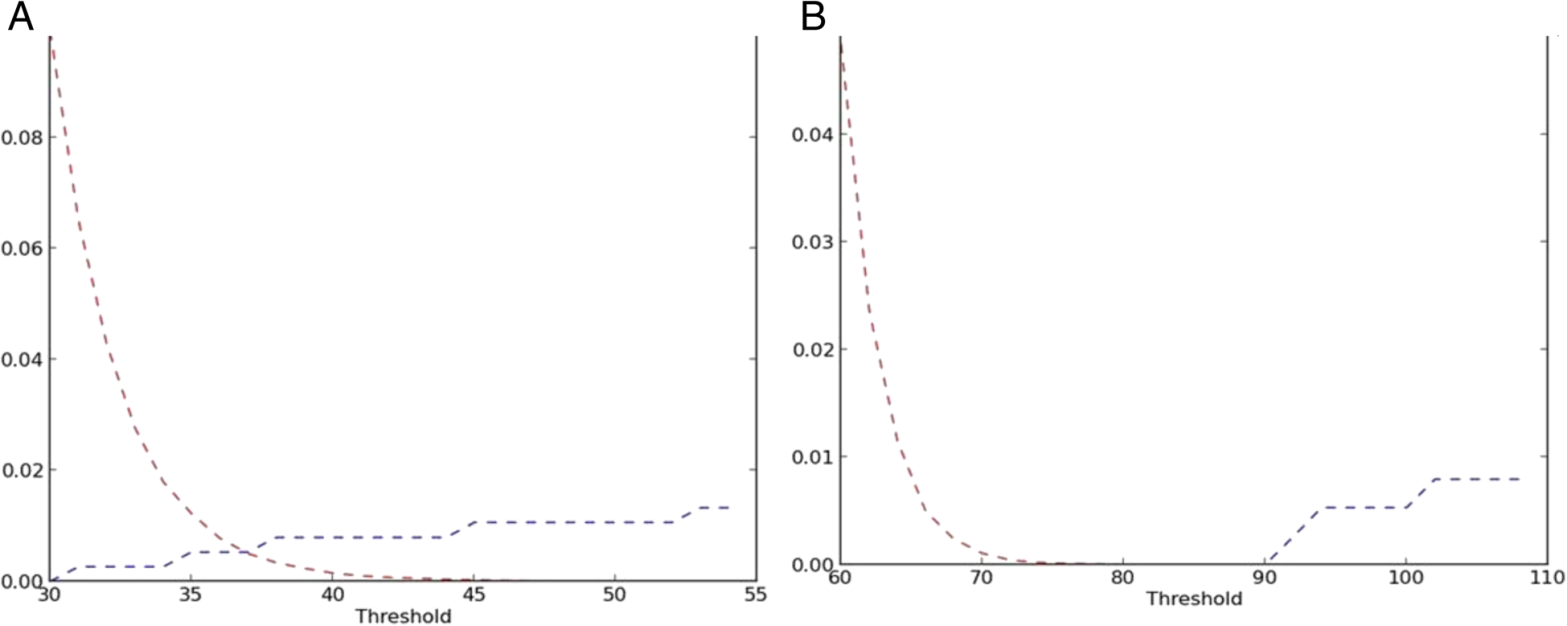
ROC plots of False Positive Matching Rate (FPMR, red) and False Negative Matching Rate (FNMR, blue) when fingerprinting RZHRG staff as a function of matching algorithm threshold. **a**. When fingerprinting left and right index fingers, the equal error rate (EER, where the FPMR and FNMR cross) is less than 1 %. **b**. When fingerprinting both index fingers and both thumbs, the ERR is zero. At a threshold of 70, the FPMR is at 1/1000 and the FNMR is 1/10,000

**Fig. 4:**
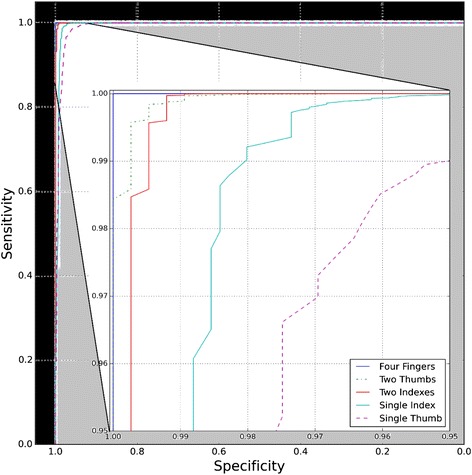
Series of receiver operator curves (ROC) for a combination of different fingers matched. A zoomed in view of the 0.95–1.0 range of sensitivity and specificity is shown

Technical challenges largely concerned system and training issues (Table 1). During validation of the false fingerprint matching and false rejection rates of the system, we fingerprinted 120 RZHRG staff three times each. Despite correct fingerprinting technique, we faced challenges with both outcomes, i.e. identification numbers that were not unique and/or that were mismatching across workflows. This testing showed that collecting fingerprints from index fingers alone (Fig. 3, Panel a) was not as accurate as capturing fingerprints from both thumbs and index fingers, which gave a false fingerprint matching rate of 1/1000 and a false rejection rate of <1/10,000 (Fig. 3, Panel b). Presenting this another way, for each combination of fingers (single thumb or index, two indexes, two thumbs, two indexes and thumbs) for the 120 individuals who provided fingerprints, we filtered scores at 0.2 threshold increments. The results are presented as a series of receiver operator curves (ROC) presented in terms of sensitivity and specificity (Fig. 4). A zoomed in view of the 0.95–1.0 range of sensitivity and specificity is shown for a clearer illustration of the benefits of using multiple fingers.

**Table 1:** Summary of system and training challenges and their solutions encountered during initial pilot testing

System issues	Resolution
Occasional MTN GSM network interruptions	Data is cached if a mobile connection is interrupted or unavailable. Cached data is uploaded to the central server after cellular reconnection is established or via wifi.
Ease of use	Resolved by Biometrac – software will be further refined given user feedback.
Device crashing and poor USB connectivity	Biometrac worked with Lumidigm to resolve the driver and USB connectivity issues and facilitate error handling (users no longer have to restart the table upon crash; they can simply disconnect and reconnect the device).
Matching	A new fingerprinting templating engine was implemented in August of 2013. Matching issues appear resolved, and we will continue to monitor false positive and false negative error rates.
Prepaid airtime overruns	Moved to postpaid airtime.
Training issues	Resolution
Differences in user technological competence	Subsequent training are incorporating local staff – pilot showed this to be effective
Lack of training among all staff	Additional training and implementation of training-of-trainers model
Some clinics forget to charge the device	Additional training

### Acceptability and barriers to uptake

We trained over 50 system users and have collected fingerprints from 111 and 42 unique FSWs in Ndola and Lusaka, respectively, since July 17, 2014. These fingerprints were collected during in-field recruitment, screening, enrollment, and follow-up. When offered electronic fingerprinting at the clinic (i.e., during screening, enrollment, or follow-up), we observed a refusal rate of 2 % (3/155 FSWs). However, among 15 women offered electronic fingerprinting in the field during pilot testing of recruitment, the refusal rate was about 50 %.

Themes identified from formative focus group work centered around confidentiality and privacy, and participants suggested that electronic fingerprinting should possibly take place in locations other than bars or on the street, that women should perhaps be recruited in groups of two or more to increase confidence and feelings of safety, that ZEHRP staff should wear badges to identify themselves and increase FSW confidence, that Queen Mothers could somehow be involved in the recruiting process to increase confidence and feelings of safety, and that incentives like lubricant and condoms should be given during the recruitment and electronic fingerprinting process.

Themes determined from the focus groups centered on a high concern about privacy while working and soliciting clients *(“I would not feel comfortable [providing E-FP in the field], because other women [in the field] would know about me”; “There are a lot of people who will become suspicious*”; *“We prefer to be fingerprinted in mobile unit, because we don’t want people to be suspicious”*), a preference for fingerprinting at home due to being too busy while working *(“Fingerprint can happen at home [in the morning] because at night [when FSWs are at the bar] everyone wanders like snake [i.e. everyone would be busy]”*); a preference for fingerprinting in a Queen Mother’s company *(“I prefer Queen Mother’s company, because she [the Queen Mother] is aware of what I do [for living]”, “She [Queen Mother] is the one that keeps my secret”*) and support from Queen Mother’s for this idea *(“Please call us before you come to fingerprint in the field [home or mobile unit]. We can arrange accompanying the staff for fingerprinting…”; “Even our girls can feel okay, because we would have explained to them.”*), a preference for fingerprinting in the presence of women who have done it before (*“I would feel more comfortable [if ZEHRP staff were accompanied by women who have E-FP experienced], because they had gotten their fingers printed like I do, and they also know what I do [for living]”; “ I would feel comfortable, because my friends [FSWs] who I do the same work with have done it [E-FP], so I could do it also”; “Because other women [who have E-FP] already know what happens will the device”), and finally, the idea that an identifying badge worn by recruiting staff may be helpful to increase confidence (“so that I can be very sure that the one doing the fingerprint is from ZEHRP”; “Because then I know they are trustworthy”; I will be more comfortable because I have seen you [ZEHRP staff] at ZEHRP site”).*

Quantitative surveys were then develop based on these themes and delivered to 45 FSWs in Lusaka (N = 10) and Ndola (N = 35) (Table 2). Before receiving an explanation of the system and that fingerprints and stored data are confidential, 44 % of women felt uncomfortable with the system, indicating the need for in-field recruiters to carefully explain what the system is used for and that it is confidential. Most women did not prefer to be fingerprinted in bars or on the street during their working hours primarily due to privacy concerns. Provision of an electronic fingerprint was reported to be more acceptable during recruiting efforts that were in the morning hours, took place in women’s homes, in the presence of a queen mother, or in the presence of a FSW that had also been fingerprinted previously. Women reported that incentives such as condoms or chitenges (a piece of cloth often worn by women) would also increase the acceptability of the system.

**Table 2:** Responses to qualitative FSW survey regarding facilitators and barriers to providing an electronic fingerprint during study recruitment

	Number	Percent
Before receiving an explanation of the system, the client was initially ____to use the device in the field upon visual inspection only.		
comfortable	25	56 %
uncomfortable	20	44 %
Womens’ preferred location for being electronically fingerprinted during recruitment (chose top two)		
The clinic	42	47 %
Their own residence	27	30 %
Bar/club	8	9 %
Mobile units in the field	8	9 %
Street	4	4 %
Womens’ least preferred location for being electronically fingerprinted during recruitment		
Bar/club	16	36 %
Street	13	30 %
Mobile units in the field	8	18 %
Residence	5	11 %
Clinic	2	5 %
Womens’ preferred time to provide an electronic fingerprint		
Morning	28	62 %
Afternoon	10	22 %
Evening	7	16 %
Do you think the electronic fingerprint system would expose who you are and what you do to other people?		
Yes	9	20 %
No	36	80 %
Would you prefer that multiple women are recruited and asked to provide an electronic fingerprint at the same time?		
Yes	19	42 %
No, because of confidentiality/privacy	26	58 %
Would you prefer to be fingerprinted in the presence of your Queen Mother?		
Yes	33	73 %
No, because of confidentiality/privacy	12	27 %
Would you prefer to be fingerprinted in the presence of other FSWs that have been fingerprinted previously?		
Yes	36	80 %
No, because of confidentiality/privacy	9	20 %
Would you prefer that ZEHRP staff wear a badge when recruiting and asking for an electronic fingerprint?		
Yes	30	67 %
No, others may become suspicious/association of ZEHRP and HIV	15	33 %
What incentives would encourage you to speak to a ZEHRP recruiter and provide an electronic fingerprint?		
Chitenge	21	47 %
Lubricant	10	22 %
Condom	31	69 %
Chlorine	5	11 %
Soap	13	29 %
ZEHRP’s service info brochure	7	16 %

### An application: fraud and co-enrollment rates

Given the low acceptability of the system at present, FSWs are not being systematically fingerprinted in the field during recruitment. However, we are fingerprinting FSWs who come into the ZEHRP clinic for screening, enrollment, and follow-up. As FSWs are the proposed cohort for a vaccine preparedness and later vaccine trial, we are using the system to detect co-enrollment (where one woman comes to the ZEHRP clinic pretending to be two different people, likely to gain incentives) and client fraud (where two different women are associated with the same study identification number). Since July of 2014, we have detected 3/153 cases of co-enrollment (2 %) and 4/153 cases of fraud (2.6 %).

## Discussion

We found that implementation of an electronic fingerprint-linked patient tracking and data collection system was feasible in this relatively resource-limited setting, and was acceptable among FSWs in a clinic setting. However, more work needs to be done for in-field use among FSWs.

Regarding the feasibility of the system, our findings are novel in that they demonstrate the viability of a sustainable, cellular-based system. This system captures basic medical information and definitively identifies individuals over time, yet does not require a constant electrical or WiFi connection. Each of the feasibility challenges we encountered was eventually resolved by improving training protocols or through communication with the software developer. We are able to successfully use the device to identify women being screened or enrolled in a study over time, and identify cases of co-enrollment and client fraud.

This system is one of the first to attempt to overcome challenges with patient identification to improve the accuracy of data collection. Moreover, its mobility has been fully capitalized on in this implementation project to recruit a typically hard to reach, stigmatized population in Zambia at high risk of HIV. Thus, our findings have major implications for key population research and health services provision. However, regarding acceptability among FSWs, our data indicate that while perhaps less than half of FSWs are comfortable providing an electronic fingerprint in the field, there are potential avenues to explore to increase acceptability. The most common reasons for not providing a fingerprint (lack of privacy/confidentiality issues while at their venues of work and in front of potential clients, i.e., at bars in the street) could be addressed by recruiting women during less busy hours, in their own homes, or in the presence of Queen Mothers. “Snowball” recruiting (the increased rate of recruitment as additional or key subjects agree to participate) may be a promising approach, given that women would find it acceptable to provide a fingerprint in the presence of a FSW that has already done so. However, the potential for recruitment of ‘false FSWs’ would have to be considered if recruiters were paid. These avenues and provision of incentives will be explored, and we hope to use these findings to develop a recruitment protocol that overcomes barriers to longitudinal study subject identification in vulnerable, hard-to-reach populations. This will improve data quality and patient management.

Some important limitations warrant consideration. We acknowledge that the small false positive (1/1000) and false negative (1/10,000) match rate inherent in automated fingerprinting systems may cause misclassification of patient data. Security questions such as year of birth, gender, and father’s name can be added to the data input system as options to facilitate confirmation of matching without compromising identity. Indeed, future reductions of false positive and false negative rates are likely with increased use of these and other contextual factors like location and time. Moving forward, as we continue to expand the use of the electronic fingerprinting system, we may encounter issues in more rural settings where the substantially greater distance between recruitment areas and clinics and poorer mobile connectivity may pose challenges to implementation feasibility. Additionally, and especially when working with vulnerable populations, concerns about coercion are warranted. We will remain watchful for the potential that women feel coerced to provide an electronic fingerprint in the field, and will continue to provide recruiting staff with appropriate device and sensitivity training.

## Conclusions

Support for biometric linked electronic medical records is illustrated by the UNAIDS position that unique individual identifiers will “strengthen fragmented health services in countries by linking data held within facilities and enabling the flow of information across the general health system and thereby also enhancing the quality, comprehensiveness and continuity of HIV-specific services” [40]. In addition to serving as a standalone electronic medical record system, the current technology can also be used to integrate biometric identification into existing electronic medical records and mobile health platforms systems. Though we are currently developing and applying this technology in a research setting, the lessons learned in this study are more broadly applicable to the integration of patient data between HIV prevention and treatment services in resource-limited settings -- an important part of a concerted effort to improve health care quality for a range of key, vulnerable populations.

## Abbreviations

FSW: Female sex worker
RZHRG: Rwanda Zambia HIV Research Group
NIH: National Institutes of Health
GSM: Global System for Mobile
ZEHRP: Zambia Emory HIV Research Project
FPMR: False Positive Matching Rate
FNMR: False Negative Matching Rate
EER: Equal error rate
IAVI: International AIDS Vaccine Initiative
